# Molecular profiling and prognostic biomarkers in chinese non-small cell lung cancer cohort

**DOI:** 10.1186/s13000-023-01349-1

**Published:** 2023-06-10

**Authors:** Fangfang Shen, Wei Guo, Xia Song, Bei Wang

**Affiliations:** 1 Department of Respiratory Medicine, Shanxi Hospital Affiliated to Cancer Hospital, Affiliated Cancer Hospital of Shanxi Medical University, Shanxi Province Cancer Hospital, Chinese Academy of Medical Sciences, Taiyuan, 030001 China; 2 The Second Hospital, Shanxi Medical University, Taiyuan, 030001 China

**Keywords:** Non-small cell lung cancer, Next-generation sequencing, Mutation patterns, Tumor mutation burden, Survival analysis

## Abstract

**Introduction:**

Comprehensive information about the genome analysis and its prognostic values of NSCLC patients in Chinese population are still needed.

**Patients:**

A total of 117 Chinese patients with NSCLC were enrolled in this study. Tumor tissues or blood were collected and sequenced by targeted next-generation sequencing of 556 cancer related genes. The associations between clinical outcomes and clinical characteristics, TMB, mutated genes, treatment therapies were analyzed using Kaplan-Meier methods and further evaluated using multivariable Cox proportional hazards regression model.

**Results:**

A total of 899 mutations were identified by targeted NGS. The most frequently mutations included *EGFR* (47%), *TP53* (46%), *KRAS* (18%), *LRP1B* (12%) and *SPTA1* (10%). Patients with mutant *TP53*, *PREX2*, *ARID1A*, *PTPRT* and *PIK3CG* had lower median overall survival (OS) than those patients with wild-type (*P* = 0.0056, *P* < 0.001, *P* < 0.0001, *P* < 0.0001 and *P* = 0.036, respectively). Using a multivariate Cox regression model, *PREX2* (*P* < 0.001), *ARID1A* (*P* < 0.001) and *PIK3CG* (*P* = 0.04) were independent prognostic factors in NSCLC. In the patients received chemotherapy, squamous patients had a significantly longer median OS than adenocarcinoma patients (*P* = 0.011). In the patients received targeted therapy, adenocarcinoma patients had a significantly longer survival period than squamous patients (*P* = 0.01).

**Conclusions:**

Our study provided comprehensive genomic alterations in a cohort of Chinese NSCLC. We also identified new prognostic biomarkers, which could provide potential clues for targeted therapies.

**Supplementary Information:**

The online version contains supplementary material available at 10.1186/s13000-023-01349-1.

## Introduction

Lung cancer is one of the leading causes of cancer related mortality worldwide, as well as in China [1]. According to 2015 statistics, there were approximately 730,000 new cases of lung cancer in China and more than 430,000 people died from this disease [2, 3]. Lung cancer is divided into non-small-cell lung carcinoma (NSCLC) and small-cell lung carcinoma, with NSCLC accounting for the most of all of cases (approximately 85%) [4]. Despite more and more researches on different treatment strategies, the 5-year overall survival rate of patients with NSCLC is less than 18% [5], which suggests that there is still a need for new targeted therapies in NSCLC. Previous studies have shown differences in the frequency of driver genes among lung cancer patients in different countries, which affect the efficacy of targeted drugs [6–9]. Thus, it is necessary to study the genomic profiles in Chinese NSCLC patients, which can help identify specific predictive-biomarkers to promote the development of precision medicine for lung cancer treatment and prevention.

Over the last decade, next-generation sequencing (NGS) technology has increasingly used for clinical diagnosis and therapies. Lots of evidences have shown the capability in accurately capturing and identifying multiple genetic alterations of NGS, including single nucleotide variant (SNV), insertions and deletions, copy number variations (CNV) and structure variations (SV), which can significantly reduce sequencing costs, improve accuracy of detection and achieve real-time monitoring progression of tumors, with high sensitivity for detecting extremely low levels of mutation frequency [10, 11]. Many studies have used NGS to analyze variations in genes and tumor mutation burden (TMB) in solid tumors [12–15]. As a result, several important genes in lung cancer have been identified, such as *EGFR*, *ALK* and *ROS1*. *EGFR* tyrosine kinase inhibitors (TKIs) erlotinib and gefitinib were the first class of molecularly targeted agents approved by the U.S, Food and Drug Administration (FDA) in 2004, up to today, erlotinib, gefitinib and other *EGFR* TKIs such as afatinib and osimertinib are all used for the treatment of metastatic NSCLC patients whose tumors have *EGFR* exon 19 deletions or exon 21 L858R mutations [16–20]. Subsequently, *ALK* inhibitor crizotinib was the first FDA-approved targeted therapy for the treatment of *ALK*-positive advanced NSCLC patients in 2011, the second generation *ALK* TKIs ceritinib, alectinib and ensartinib were all approved for metastatic *ALK* + NSCLC [21–26]. And crizotinib was approved for metastatic *ROS1* positive NSCLC in 2016 [24, 27–29]. These discoveries have revolutionized treatment of patients whose tumor harbor these genes. Recent years, many studies have explored the prognostic values of the mutated genes which have been considered as potential important therapeutic target in NSCLC, such as *MET* [30], *KMT2D* [31] and *PIK3CA* [32], which has greatly facilitated the discovery of gene-based tumor biomarkers [33, 34]. However, until now, the knowledge of genetic variations and molecular biomarkers in NSCLC are still lacking in the Chinese population. Therefore, it is necessary to explore the genetic mutational landscape and identify prognostic biomarkers of Chinese patients to deeply understand the clinical outcomes and find new treatment options for lung cancer.

In the present study, we established a panel comprised 556 genes to detect somatic mutations in 117 samples from Chinese NSCLC patients. Furthermore, we explored the prognostic values of tumor burden mutation (TMB), clinical characteristics, gene mutations and treatment therapies. Our study aimed to provide a comprehensive genomic profiling of 117 Chinese patients with NSCLC, and provide new prognostic biomarkers to help find new therapeutic targets.

## Patients and methods

### Patient cohort and DNA extraction

Tumor tissues or blood were obtained from 117 Chinese patients with NSCLC at Shanxi Cancer Hospital between 2019 and 2022. The diagnosis of all the samples in the cohort was performed on the morphology of hematoxylin & eosin staining (HE) by two experienced molecular pathologists and the content of tumor cell (tumor purity) was higher than 50%. 8–10 of 5–10 μm tumor slices of formalin-fixed paraffin-embedded (FFPE) of tumor tissues and 8–10 mL plasma samples from patients were collected for further use [35]. Genomic DNA was isolated from tissues or blood using the DNeasy Blood and Tissue Kit (69,504, QIAGEN, Venlo, Netherlands). DNA content was determined by Agilent 2100 Bioanalyzer (USA). Libraries were constructed if the gDNA amount from the tumor tissue/plasma samples ≥ 200 ng [35]. All patients signed an informed consent before joining the study. Demographic and clinical characteristics were collected from patients. This study was approved by the Ethics Committee of Shanxi Cancer Hospital and performed in accordance with the World Medical Association Declaration of Helsinki (as revised in 2013).

### Next-generation sequencing

Next-generation sequencing (NGS) performed on ZhenXinan ctDNA NGS Panel (Tongshu BioTech, Shanghai, China). Sequencing libraries of different components were prepared using the KAPA Hyper Prep kit (KAPA Biosystems) with an optimized manufacturer’s protocol. Enriched libraries were amplified and detected on Illumina Novaseq 6000 platform (Illumina) in accordance with manufacturer’s instructions. The average sequencing depth in tissues is ≥ 1000×; the average sequencing depth in plasma cfDNA is ≥ 7000×. The variant allele frequency (VAF) is ≥ 1% for tissue DNA and ≥ 0.1% for cfDNA from plasma [35]. The sequencing data in the FASTQ format were mapped to the human genome (hg38) using BWA aligner 0.7.10. Local alignment optimization, variant calling and annotation were performed using GATK 3.2 (https://gatk.broadinstitute.org/), MuTect [36] and VarScan [37] respectively. Somatic mutations existing in at least 2 of the results of the 3 software were selected as high confident mutations and to be involved in the further bioinformatics and biostatistical analysis.

### Statistical analysis

Oncoplot mutations were plotted by using the MAfTools R package. Mutations in cancer-related driver genes were also analyzed. The clusterProfiler R package was used to visualize the Gene Ontology (GO) and Kyoto Encyclopedia of Genes and Genomes (KEGG) pathway enrichment results of mutated genes in all the samples [38]. Fisher’s exact test was used to analyze the associations between two groups. Overall survival (OS) was defined as the time from the date of diagnosis to the date of death or the last follow-up visit for patients. The definition of progression-free survival (PFS) was the time from start of treatment to the clinical or radiographic progression, or the end of follow-up. Median OS and PFS were calculated using Kaplan–Meier method and survival curves were compared with log-rank tests. The variables putatively associated with patient survival were analyzed with the log-rank test and Cox proportional hazards model. The statistical software package SPSS 22.0 was used for statistical analysis. All tests were bilateral, with *P* values < 0.05 indicating significant statistical difference.

## Results

### Clinicopathological characteristics of NSCLC patients

A total of 117 patients were included in this study. The clinical and pathological information of all patients in the cohort are summarized in Table 1. A total of 72 (61.5%) patients are males, and 60 patients (51.3%) presented a smoking history. The median age of the patients was 61 years (range, 28–78 years). Most of the patients showed metastases to the lungs and/or other sites (78/117, 66.7%). Regarding histological subtypes, lung adenocarcinoma was the most common subtype (96/117, 82.1%), followed by lung squamous cell (15/117, 12.8%). 8 patients (6.8%) were classified as stage I and II, and 109 patients (93.2%) as stage III and IV. Males were especially prevalent in late-stage (III-IV) group. In all patients, 44 (37.6%) received targeted therapy. A total of 55 patients (47.0%) received chemotherapy; of these, 36 (65.5%) underwent chemotherapy alone, 14 (25.5%) underwent chemotherapy and immunotherapy, while the remaining patients received chemotherapy and antivascular therapy.

**Table 1:** Clinical characteristics of patients with NSCLC included in this study

	N (%)
**Total**	117 (100)
**Age**	
< 70	100 (85.5)
≥ 70	17 (14.5)
**Gender**	
Male	72 (61.5)
Female	45 (38.5)
**Smoking**	
Never smoke	57 (48.7)
Current or former	60 (51.3)
**Metastasis**	
Yes	78 (66.7)
No	39 (33.3)
**Histology**	
Squamous	15 (12.8)
Adenocarcinoma	96 (82.1)
Other	6 (5.1)
**Stage at diagnosis**	
I	6 (5.1)
II	2 (1.7)
III	17 (14.5)
IV	92 (78.6)
**First-line therapy**	
Targeted therapy	44 (37.6)
Chemotherapy	55 (47.0)
Other	18 (15.4)

### Somatic mutation landscape in NSCLC patients

**Fig. 1:**
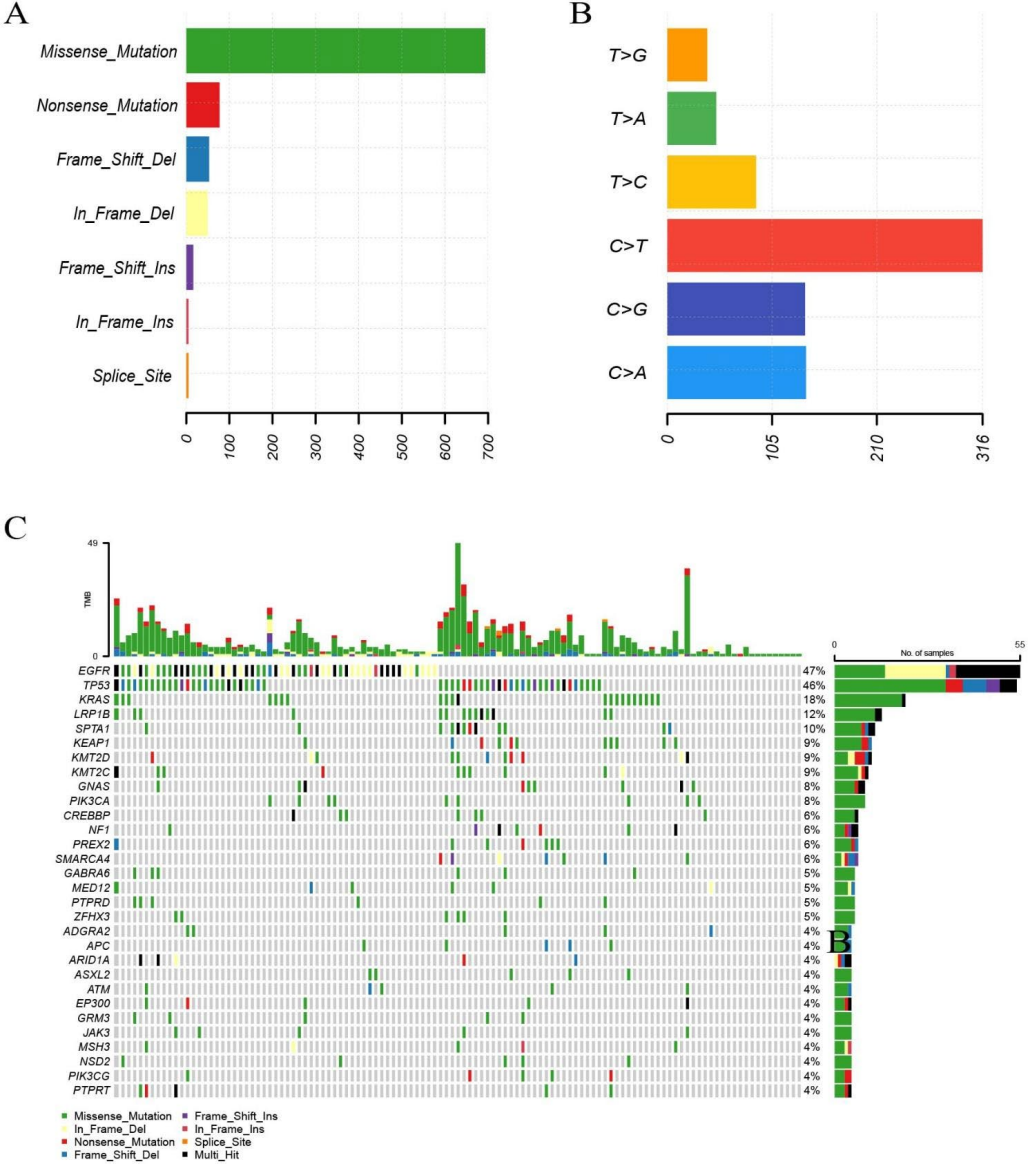
Genetic alterations of the 117 NSCLC patients. (A) Distribution of gene mutation types of all the samples. (B) Single mutation types of all the samples. (C) Overview of the 30 genes with the highest mutation frequency

NGS results showed that different somatic mutations occur in all genes, including amplification and fusion, chromosomal structural variation, insertion and deletion, and point mutation. In the 117 samples tested, a total of 899 mutations were identified, and the dominant mutation type was missense mutation (693/899, 77.1%; Fig. 1A). Variant spectrum showed that C > T had the highest mutation percentage in all patients (Fig. 1B). The distribution of top 30 mutations is shown in Fig. 1C. The top 10 most frequently mutated genes in all the samples were *EGFR* (55/117, 47%), *TP53* (54/117, 46%), *KRAS* (21/117, 18%), *LRP1B* (14/117, 12%), *SPTA1* (12/117, 10%), *KEAP1* (11/117, 9%), *KMT2D* (11/117, 9%), *KMT2C* (10/117, 9%), *GNAS* (9/117, 8%) and *PIK3CA* (9/117, 8%). The number of patients with *EGFR* and *TP53* mutations was basically the same (47% and 46%, respectively), of these patients, 26 had *EGFR* and *TP53* co-mutations (47.3% of patients with *EGFR* and 48.1% of patients with *TP53*). Then, we further subdivided top 10 gene mutations into exons, the results are shown in supplementary Fig. 1.

CNV analysis showed that 394 genes had copy number amplification (supplementary Fig. 2A). Among these, *EVI2A* (82/117, 70.09%), *EVI2B* (82/117, 70.09%), *NF1* (82/117, 70.09%), *OMG* (82/117, 70.09%) and *BRAC1* (72/117, 61.54%) were the genes with highest amplification. A total of 830 genes had copy number deletion (supplementary Fig. 2B). The genes with the highest copy number deletions were *KAT6A* (76/117, 64.96%) and *RASA1* (72/117, 61.54%).

### GO and KEGG enrichment analysis

Next, we performed GO and KEGG enrichment analysis on top 30 mutated genes. GO enrichment analysis is divided into molecular function analysis (MF), biological process analysis (BP) and cellular component analysis (CC). As shown in supplementary Fig. 3A, the results showed that the top five biological process are response to radiation, response to light stimulus, phosphatidylinositol 3-kinase signaling, somitogenesis and cellular response to heat. At the molecular function level, these genes are significantly enriched in RNA polymerase II-specific DNA-binding transcription factor binding, DNA-binding transcription factor binding, transcription coactivator activity, transcription coregulator activity and p53 binding (supplementary Fig. 3B). The top five cellular components are extrinsic component of membrane, cytoplasmic side of plasma membrane, extrinsic component of plasma membrane, npBAF complex and Wnt signalosome (supplementary Fig. 3C). KEGG analysis revealed that these genes are mainly concentrated in human papillomavirus infection, microRNAs in cancer, hepatocellular carcinoma, phospholipase D signaling pathway, colorectal cancer and other signaling pathways (supplementary Fig. 3D).

### Relationships between TMB and clinicopathological characteristics

**Fig. 2:**
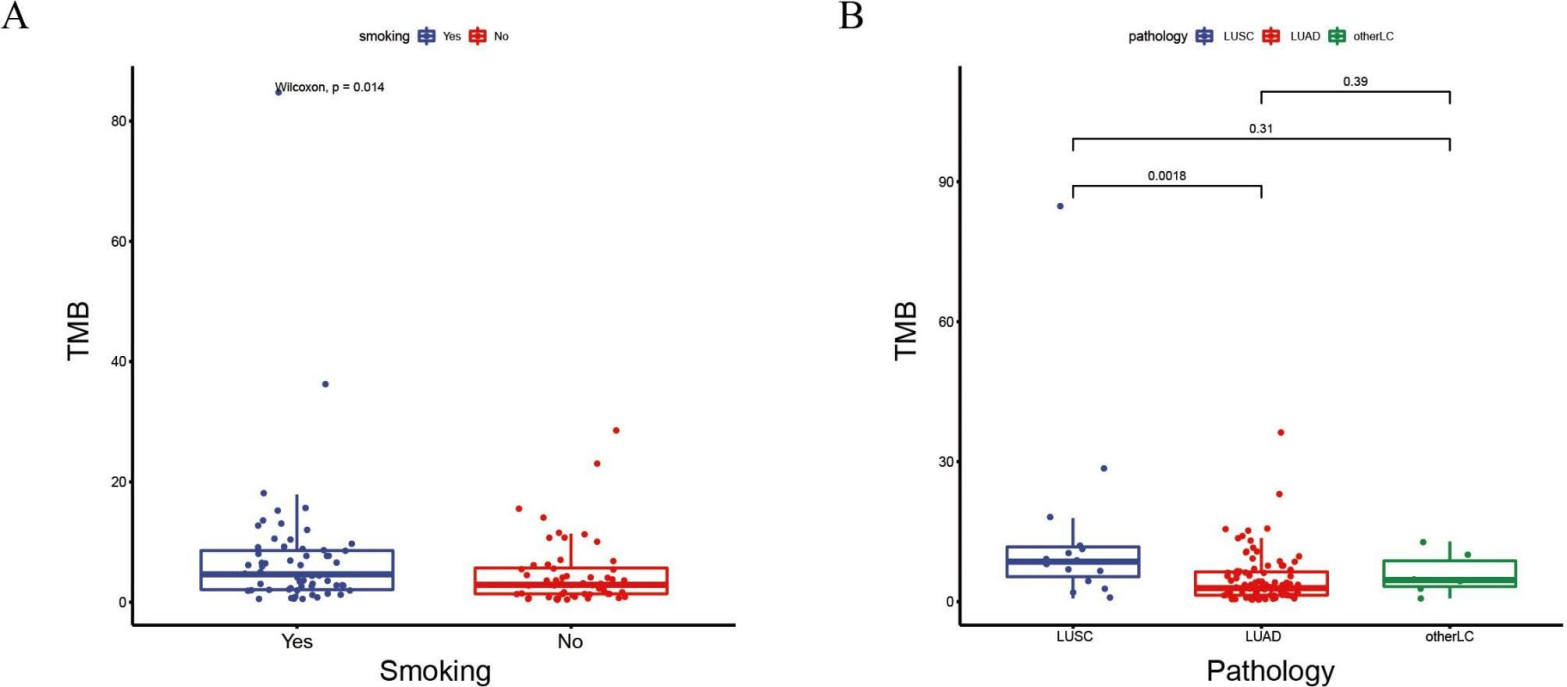
TMB analysis of the 117 NSCLC patients. (A) Association between TMB and smoking status. (B) Association between TMB and tumor types (OtherLC: Other Lung cancer types)

Tumor mutation burden (TMB) has been regarded as a biomarker to predict immunotherapy response in clinical oncology, including NSCLC. We performed comparative analysis of the clinical characteristics to explore the association between TMB and Chinese NSCLC patients. Somatic mutations were obtained after removing splicing mutations, and the valid TMB values were obtained after dividing the number of somatic mutations by the size of the panel. Wilcoxon Signed Rank Test was used to analyze the differences of TMB in clinical characteristics. There were significant differences in TMB between smokers and nonsmokers (median, 4.65 Mutations/Mb and 2.9 Mutations/Mb, respectively; *P* = 0.014; Fig. 2A). The median TMB of squamous is 2.97 times that of adenocarcinoma (median, 8.6 Mutations/Mb and 2.9 Mutations/Mb, respectively). Significant difference was observed between these two tumor types (*P* = 0.0018; Fig. 2B). There were no significant differences in TMB between younger patients and elder patients (range, 0.7–84.9 Mutations/Mb; median, 3.6 Mutations/Mb; *P* = 0.30), males and females (median, 4.3 Mutations/Mb and 2.9 Mutations/Mb, respectively; *P* = 0.10), patients with metastasis and without metastasis (2.9 Mutations/Mb and 5.0 Mutations/Mb, respectively; *P* = 0.21), and early-stage and late-stage patients (median, 1.75 Mutations/Mb and 3.6 Mutations/Mb, respectively; *P* = 0.065).

### Relationships between mutations and clinicopathological features

We compared clinicopathological characteristics of the cohort of patients with the top 30 mutated genes (supplementary Table 1). The results showed that the *EGFR* mutation rate was significantly higher in females (30/45, 66.7%) than in males (25/72, 34.7%; *P* = 0.0011), in never smokers (35/57, 61.4%) than in smokers (20/60, 33.3%; *P* = 0.0030), in patients with metastasis (44/78, 56.4%) than in those without metastasis (11/39, 28.2%; *P* = 0.0057), and in patients with adenocarcinoma (51/96, 53.1%) than in those with squamous (3/15, 20%; *P* = 0.025). In contrast, the *KRAS* mutation rate was higher in males (17/72, 23.6%) than in females (4/45, 8.9%; *P* = 0.050). In addition, the *KRAS* mutation rate was higher in smokers (14/60, 23.3%) than in never smokers (7/57, 12.3%; *P* = 0.15), in patients with squamous (3/15, 20%) than in those with adenocarcinoma (18/96, 18.8%; *P* = 1), and in patients with early-stage (2/8, 25%) than in those in late-stage (19/109, 17.4%; *P* = 0.63). However, there were no significant differences between *KRAS* mutations with histological subtypes, smoking status and the stage. *KEAP1* mutations were only occurred in males (11/72, 15.3%), which is significantly more frequent than in females (0%; *P* = 0.0064); and the *KEAP1* mutation rate was significantly higher in smokers (10/60, 16.7%) than in never smokers (1/57, 1.8%; *P* = 0.0085), and in more prevalent among patients in early stage (3/8, 37.5%) than in those in late stage (8/109, 7.3%; *P* = 0.027). Besides, *PREX2* mutations were only occurred in males (7/72, 9.7%), which is significantly more frequent than in females (0%; *P* = 0.042); *PREX2* mutations were only occurred in smokers (7/60, 12%), which was significantly higher in never smokers (0%; *P* = 0.013), and *PREX2* mutations were significantly higher in patients with squamous (4/15, 26.7%) than those with adenocarcinoma (2/96, 2.1%; *P* = 0.0029). In addition, the *NSD2* mutation rate was significantly higher in elder patients (3/17, 17.6%) than in younger patients (2/100, 2%; *P* = 0.022). The *KMT2D*, *GNAS*, *EP300*, *GRM3*, and *PIK3CG* mutations were all significantly higher in patients with squamous than those with adenocarcinoma (*P* = 0.0066, *P* = 0.019, *P* = 0.0075, *P* = 0.017, and *P* = 0.0075, respectively). The *KMT2C* mutation rate was significantly more frequent in patients without metastasis (7/39, 17.9%) than those with metastasis (3/78, 3.8%; *P* = 0.015). No association was found between *TP53*, *LRP1B*, *SPTA1* and clinicopathological characteristics.

### Survival analysis

#### The relationship between TMB and prognosis

**Fig. 3:**
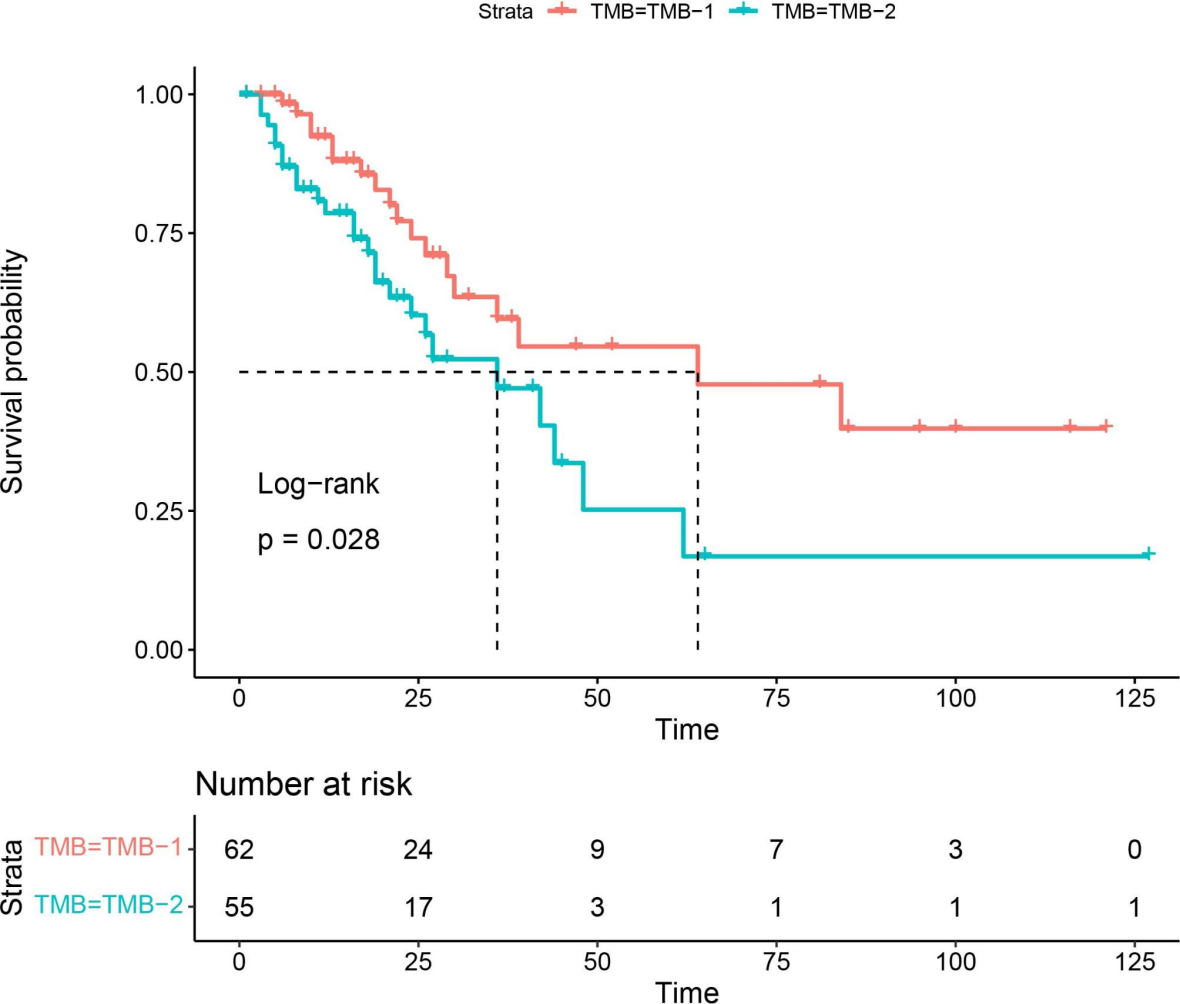
Overall survival analysis of high TMB (> 3.6 Mutations/Mb) and low TMB (≤ 3.6 Mutations/Mb).

Nonsynonymous TMB was calculated and analysis was performed to assess the effect of TMB on prognosis. We used the median TMB (3.6 Mutations/Mb) as the cut-off value to divide TMB into two groups, TMB ≤ 3.6 Mutations/Mb as TMB-1 group and TMB > 3.6 Mutations/Mb as TMB-2 group. Univariate analysis showed that OS was significantly longer in patients with lower TMB than those patients with higher TMB (64 months vs. 36 months, *P* = 0.028) (Fig. 3). However, multivariate analysis showed that TMB was not an independent prognostic factor (*P* = 0.49; supplementary Table 1).

### Effects of clinicopathological features on overall survival

The average follow-up was for 25.7 months, and the median OS was 18 months (range, 1-127 months). As shown in supplementary Fig. 4, patients with squamous has a significantly longer survival period than those patients with adenocarcinoma and other lung cancer types (median survival time, 62 months vs. 39 months vs. 11 months; *P* < 0.001, by log-rank test), and there were no significantly correlations between other clinicopathological features and OS (supplementary Table 1).

### Effects of genomic alterations on overall survival

**Fig. 4:**
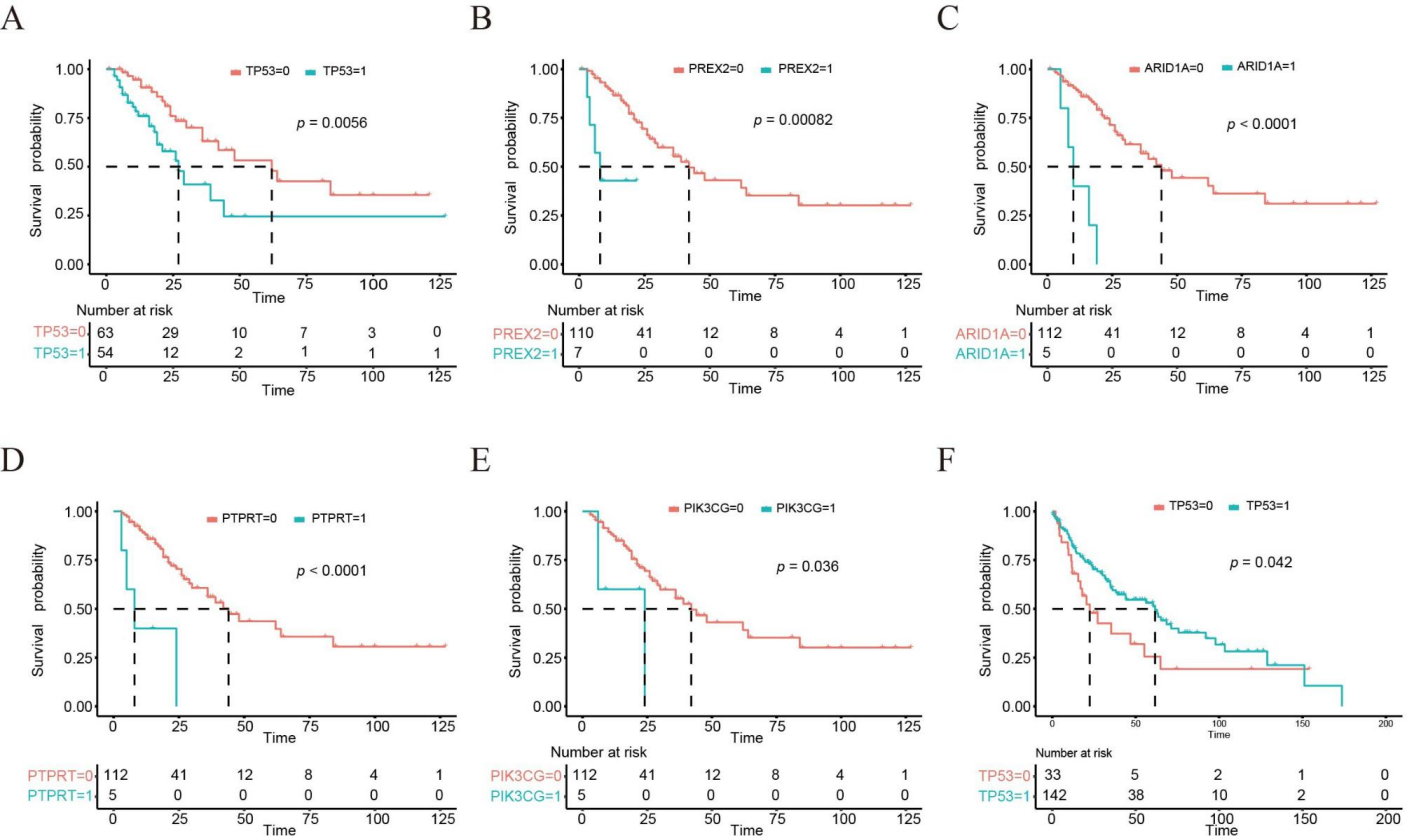
Overall survival analysis of the mutated Genes. (A) Kaplan-Meier curve of *TP53* mutant. (B) Kaplan-Meier curve of *PREX2* mutant. (C) Kaplan-Meier curve of *ARID1A* mutant. (D) Kaplan-Meier curve of *PTPRT* mutant. (E) Kaplan-Meier curve of *PIK3CG* mutant. (F) TCGA validation between *TP53* mutant and overall survival in lung squamous cohort

Patients with *TP53* (median survival time, 27 months vs. 62 months; *P* = 0.0056), *PREX2* (median survival time, 8 months vs. 42 months; *P* < 0.001), *ARID1A* (median survival time, 10 months vs. 44 months; *P* < 0.0001), *PTPRT* (median survival time, 8 months vs. 44 months; *P* < 0.0001) and *PIK3CG* (median survival time, 24 months vs. 42 months; *P* = 0.036) mutations survived for a significantly shorter period than those without the mutations, suggesting that these mutations predict positive factors for NSCLC prognosis (Fig. 4A-E). In addition, we further validated these five prognosis-related genes in the cohort of lung adenocarcinoma and lung squamous from the Cancer Genome Atlas (TCGA) data set. In the squamous patients from the TCGA database, the *TP53* mutation was significantly associated with prognosis (Fig. 4F). Other four genes had no significantly associations with prognosis in lung adenocarcinoma or/and lung squamous. In contrast, patients with *KRAS* mutations survived longer than those without mutations (median survival time, 64 months vs. 39 months), whereas there was no significant difference (*P* = 0.45).

**Fig. 5:**
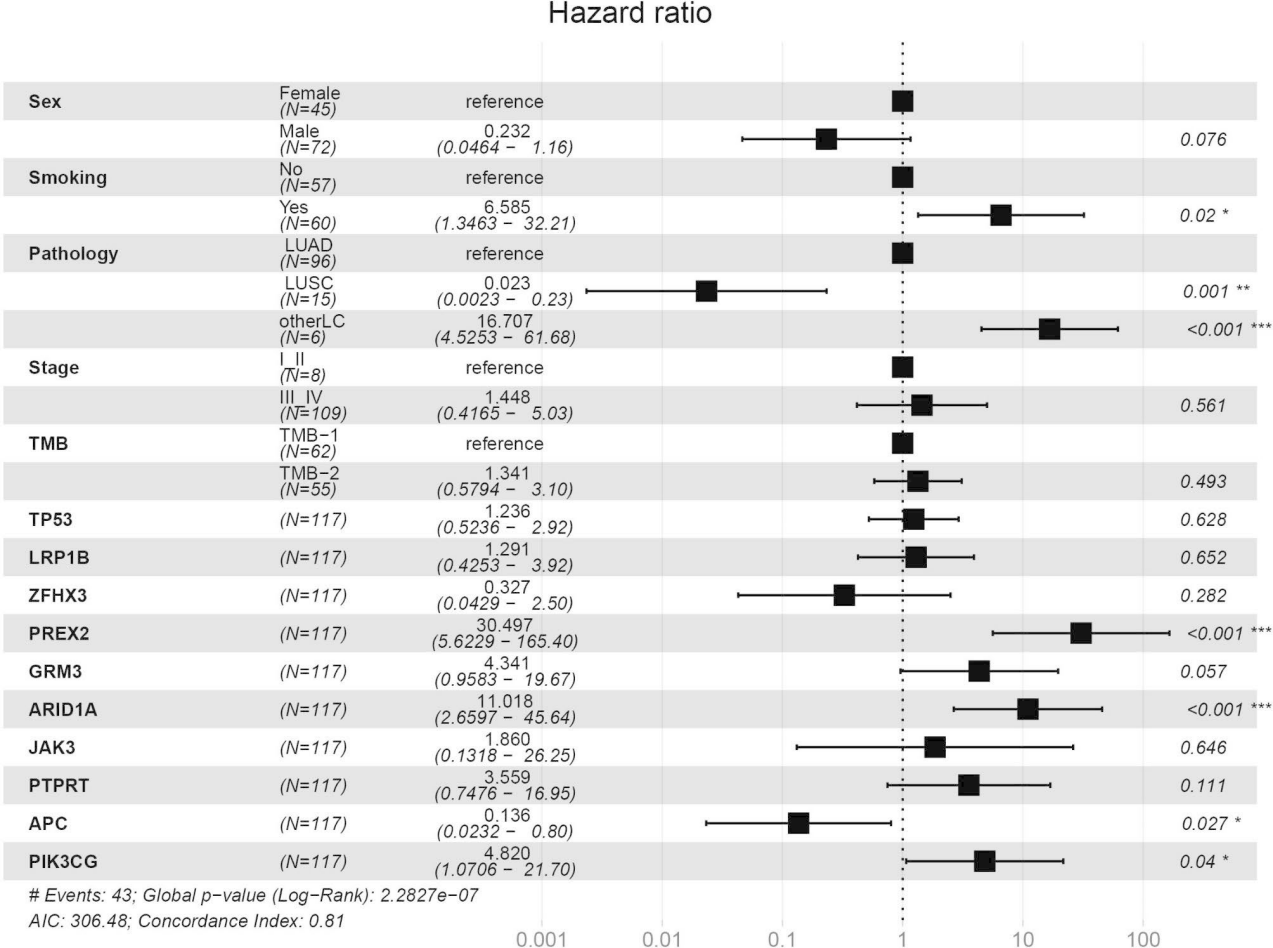
Multivariate analysis between mutations and overall survival

Multivariate Cox proportional hazard analyses of clinicopathological features showed that smoking (*P* = 0.02) and the histological subtype (*P* < 0.001) were independent prognostic factors for overall survival (Fig. 5). In addition, the results demonstrated that the mutated gene *PREX2* (*P* < 0.001), *ARID1A* (*P* < 0.001) and *PIK3CG* (*P* = 0.04) were independent prognostic factors (Fig. 5). However, *TP53* and *PTPRT* were not independent prognostic factors (Fig. 5; supplementary Table 1).

### Effects of treatments on progression-free survival

**Fig. 6:**
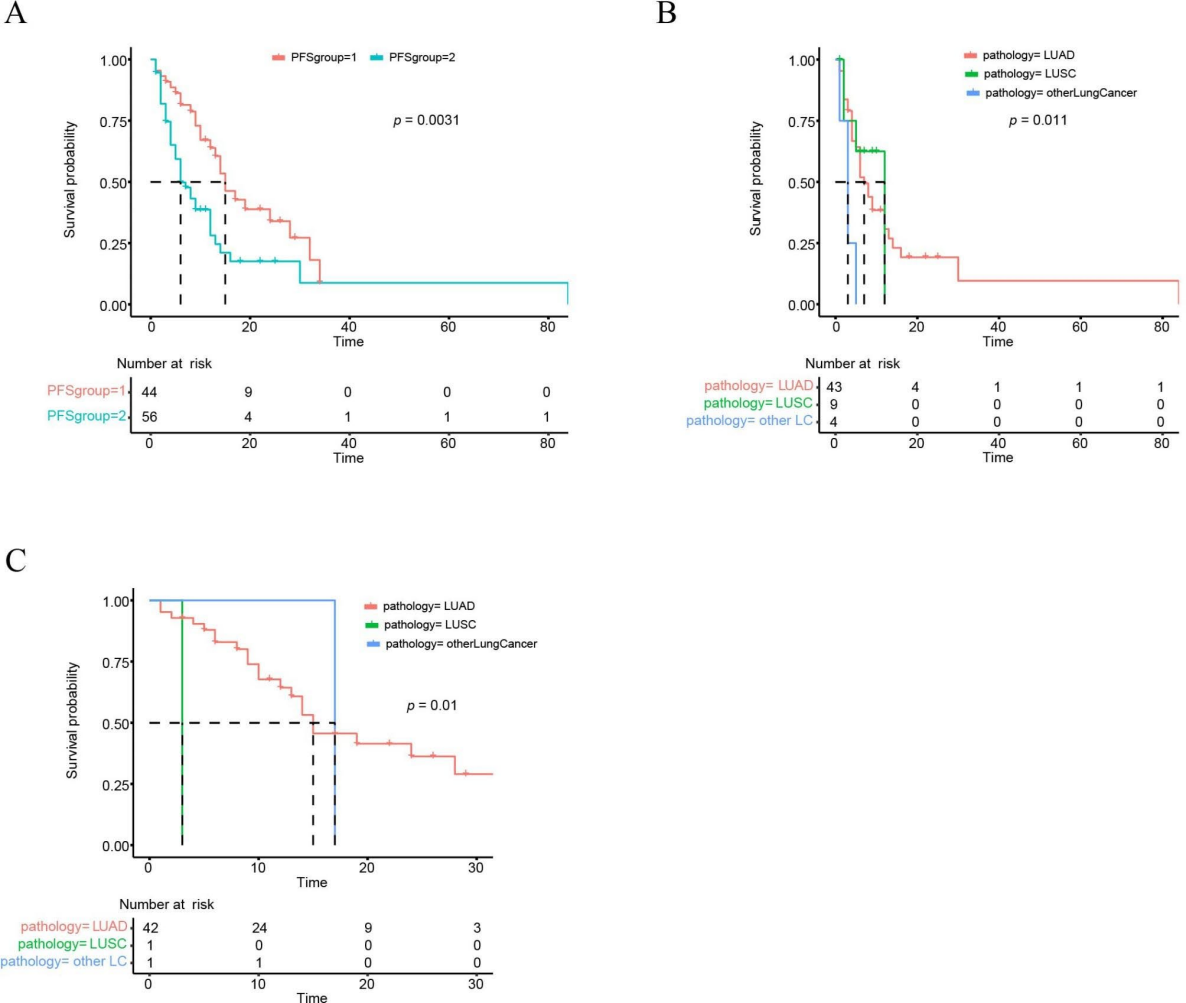
Progression-free survival analysis of treatments. (A) Kaplan-Meier curve of the patients received chemotherapies and targeted therapies. (B) Kaplan-Meier curve of different pathological types of the patients who received chemotherapies. (C) Kaplan-Meier curve of different pathological types of the patients who received targeted therapies

Univariate analysis revealed that the patients underwent targeted therapy (PFS group 1) had a significantly prolonged PFS as compared to those with chemotherapy (PFS group 2) (15 months vs. 6 months; *P* = 0.0031; Fig. 6A). In the patients who underwent chemotherapies, squamous patients had a significantly longer PFS than adenocarcinoma patients (12 months vs. 7 months; *P* = 0.011; Fig. 6B). In the patients who underwent targeted therapies, adenocarcinoma patients had a significantly longer PFS than squamous patients (15 months vs. 3 months; *P* = 0.01; Fig. 6C).

## Discussion

In this study, we used a targeted NGS panel to perform comprehensive genomic profiling on tumor specimens from 117 Chinese NSCLC patients. We identified 899 somatic mutations by 556-genes panel. The most frequently mutated genes were obtained. Notably, *EGFR* and *TP53* were co-mutated in most sample. 394 genes and 830 genes showed copy number amplification and copy number deletion, respectively. The top 30 mutated genes were specifically enriched in the cancer-associated pathways. Then, we analyzed the association between TMB and clinical characteristics. There were significant differences in TMB between the smoking status and pathological subtypes. Similar results were shown in previous studies. In addition, we explored the relationship between clinical characteristics and driver alterations. Finally, we performed survival analysis, including the relationships between TMB and overall survival, mutated genes and overall survival, treatments and progression-free survival. We have identified genes whose mutations are significantly associated with survival in NSCLC and further confirmed these mutated genes in the TCGA database.

Based on the targeted NGS, potentially genetic alterations were detected in 87.18% of Chinese NSCLC patients in the present cohort, which was similar to the previously study [39]. We determined the most frequently mutated genes in the NSCLC patients, including *EGFR*, *TP53*, *KRAS* and *LRP1B*. These mutated genes have been reported in NSCLC previously [40–42]. The distribution of genetic alterations in the Chinese patients showed differences with those in the Caucasian patients. Compared with a study on the American population, their results showed that the most frequently mutated gene is *KRAS*, followed by *EGFR* [43]. Interestingly, in a study of Chinese NSCLC, the highest mutated genes were *TP53*, *EGFR* and *CREBBP* [3]. However, our results showed certainly consistency with those in some studies. In a study on the Chinese patients, the most frequently mutated genes were *EGFR*, *TP53*, *KRAS* and *ALK* [15]. In a study on the East Asian patients, the most frequently mutated genes were *EGFR*, *TP53*, *ALK* and *KRAS* [44]. *EGFR* was the most frequently mutated gene in both this study and our study, accounting for about 47% in all the samples.

Asian population have unique clinical features and tumor histology, exhibiting different prevalence of oncogenic mutations [45]. Association between driver genes and clinical features were consistent with prior reports. For example, *EGFR* mutations were more common in females, never smokers and in patients with adenocarcinoma [41, 46]. In addition, it was more common in early-stage patients than in those with late-stage, which was not found in the study of Liu’s et al [44]. The *KRAS* mutations were also more common in males than females [47]. Besides, we found other two driver alterations that associated with clinical characteristics. *KEAP1* mutations were only occurred in males, enriched in smokers than never smokers, and in early-stage patients than in late-stage patients. According to the previous studies, approximately 20–30% of lung adenocarcinoma harbor the *KEAP1* mutations which correlate with poor prognosis [42, 48, 49]. Romero et al’ s study provided the evidence for stratification of patients with *KEAP1* mutation as possible responders to targeted *SLC33A1* inhibition [50]. Saleh et al’ s study showed that *KEAP1* mutations accounted for about 17% of NSCLC patients, and *KEAP1* mutation was significantly associated with higher age, male sex, adenocarcinoma differentiation and advanced stage, and also represented an independently negative prognostic biomarker [51]. *PREX2* mutations only occurred in males and smokers, and *PREX2* mutations were significantly higher in patients with squamous than those with adenocarcinoma. In Wang et al’ s study, *PREX2* mutations were found to be highly frequent in patients with multiple primary lung adenocarcinoma than those with single primary lung adenocarcinoma (*P* = 0.0456).

In terms of clinical outcomes, patients with low TMB and adenocarcinoma were associated with better overall survival. Patients with *TP53*, *PREX2*, *ARID1A*, *PTPRT* and *PIK3CG* had worse prognosis. *TP53* was commonly considered as a prognostic factor with poor prognosis of lung cancer in many studies [52, 53]. However, opposite or neural results were also reported [54, 55]. We found that patients with *TP53* mutations had better survival than those with wild type in TCGA cohort of lung squamous patients, which is contrast with our results. Since most of the patients are lung adenocarcinoma in our study, which could explain these results. *PREX2* mutations were frequently occurred in melanoma [56] and were considered as new candidate drivers of pancreatic carcinogenesis [57]. Previous studies have found that *ARID1A* mutations are likely to be related to the higher immune infiltrates in endometrial cancer, stomach cancer and colon cancer [58]. The study of Zhu et al. suggested that *ARID1A* mutations were related to the good prognosis of immune checkpoint inhibitors (ICI) therapy based on the pan-cancer population [59]. The study of Chen et al. suggested that *PTPRT* mutation is associated with poor progression-free survival in pan-cancer and NSCLC [60]. Contrary to our results, the study of Zhang et al. showed that melanoma patients with *PTPRT* mutations harbored a significantly elevated ICI response rate and a prolonged survival outcome, and in the NSCLC cohort, the favorable response and immunotherapy survival were also observed in *PTPRT*-mutated patients [61]. The study of the association between *PIK3CG* and prognosis is still lacked. In a study of Wu et al., *PIK3CG* considered as favorable prognostic factor [62]. In multivariate analysis accounting for gender, smoking status, pathology, stage, TMB and the mutated genes with potential prognostic values, *ARID1A*, *PREX2* and *PIK3CG* retained the significant independent prognostic factors. However, for *TP53* and *PTPRT*, the survival impact of mutations as independent variations did not remain significant. There were also no significant differences in overall survival between the patients with *EGFR*, *KRAS* mutations and those without these genes in this study. However, recent advances of studying *KRAS* biology have led the discovery of *KRAS* p.G12C-specific inhibitors which show the great promising clinical results [63]. In a phase I trial (NCT03600883), sotorasib showed anticancer activity in advanced NSCLC patients who harbored *KRAS* p.G12C mutation with a median follow-up of 11.7 months [64]. Furthermore, in a phase II trial (NCT03785249), adagrasib also showed clinical efficacy without new safety signals in NSCLC patients with advanced or metastatic tumors harboring *KRAS* p.G12C mutation [65]. As the *KRAS* p.G12C mutation is the most frequent variation among all *KRAS* mutations in NSCLC, presenting in 10–13% of patients with lung adenocarcinomas, the potentially effective therapies of *KRAS* p.G12C mutant NSCLC may help to optimize clinical treatment outcomes in this important and common subtype of lung cancers [63, 66].

Targeted therapy has led an important impact in lung cancer management and clinical outcomes over the past two decades. In the present cohort, patients received targeted therapy had a better PFS than those received chemotherapies. In addition, we found that in the patients that received chemotherapies, squamous patients had a better PFS than adenocarcinoma patients, and in the patients that received targeted therapies, adenocarcinoma patients had a better PFS than squamous patients. Adenocarcinoma represents 50–60% of total NSCLC cases, whereas squamous cell carcinoma represents 20–30% [67]. Most prevalent actionable mutations in adenocarcinoma include *KRAS*, *EGFR*, *ALK*, *RET*, *ROS1*, *BRAF*, *HER2*, *MET* [42, 68–70]. However, most of these mutations are rare in squamous NSCLC, so there were much more adenocarcinoma patients who could receive targeted therapies than squamous patients, which could explain the above results.

## Conclusion

In this study, we assessed the mutation profiles, copy number variations and tumor mutation burden of 117 NSCLC patients. There were significant differences in TMB between smokers and nonsmokers, and between patients with adenocarcinoma and those with squamous. However, TMB was not associated with other clinical characteristics. Patients with low TMB had significantly longer survival period than those with high TMB. Our discovery suggested that mutant *TP53*, *PREX2*, *ARID1A*, *PTPRT* and *PIK3CG* considered as prognostic factors with poor prognosis of NSCLC. Adenocarcinoma patients had a significantly longer survival period than squamous patients who received targeted therapies. Our findings indicated that targeted NSG panel is a good tool for tumor molecular characterization. In addition, our results were expected to provide implications for cancer translational research and management of NSCLC.

## Electronic supplementary material

Below is the link to the electronic supplementary material.

Supplementary Fig. 1: Distribution of the mutations in the top 10 mutated genes.

Supplementary Fig. 2: Copy number variations of the top 30 mutated genes. (A) Distribution of the copy number amplification. (B) Distribution of the copy number deletion.

Supplementary Fig. 3: Go and KEGG enrichment analysis of the top 30 mutated genes. (A) Biological process analysis. (B) Molecular function analysis. (C) Cellular component analysis. (D) KEGG enrichment analysis.

Supplementary Fig. 4: Overall survival analysis of the tumor type.

Supplementary Table 1: Multivariate Cox proportional hazard analyses of clinicopathological factors for OS in NSCLC cohort.

Supplementary Table 2: Relationship between top 30 gene mutations and clinicopathological features.
