# Supplementary figures and images for: Molecular profiling and prognostic biomarkers in chinese non-small cell lung cancer cohort
Source: Diagn Pathol. 2023 Jun 10;18:71. doi: 10.1186/s13000-023-01349-1 (PMC10257305; doi:10.1186/s13000-023-01349-1)

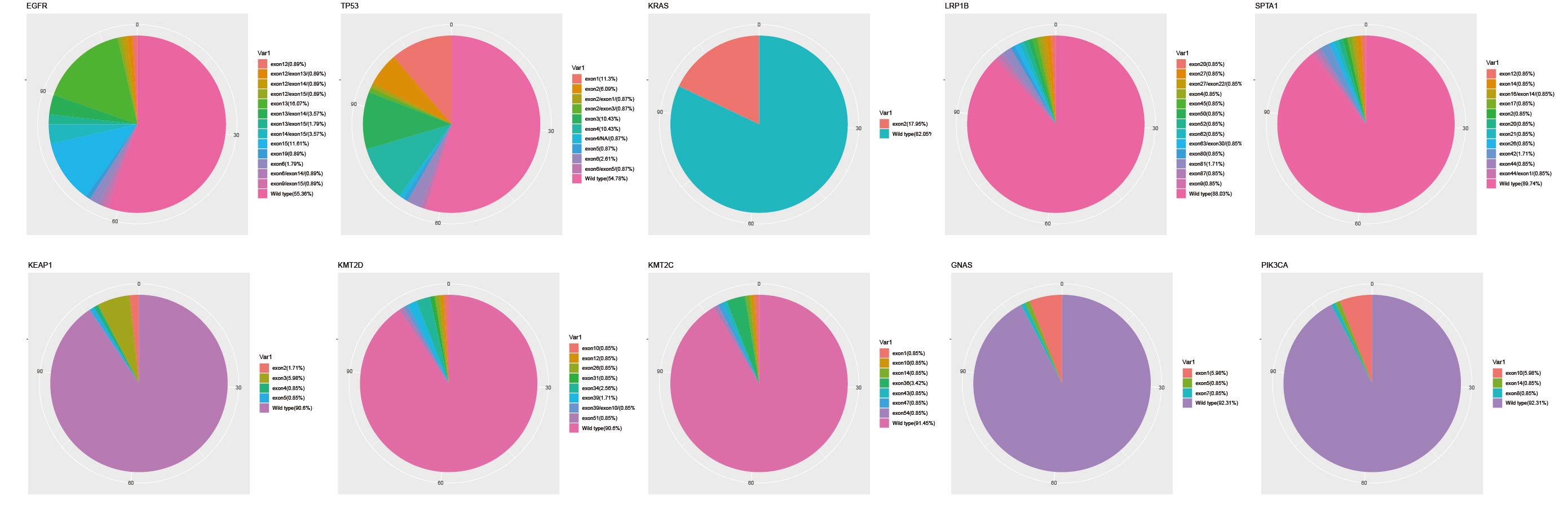

Supplement: Supplementary file 1 — Supplementary Fig. 1: Distribution of the mutations in the top 10 mutated genes. [file 13000_2023_1349_MOESM1_ESM.jpg]

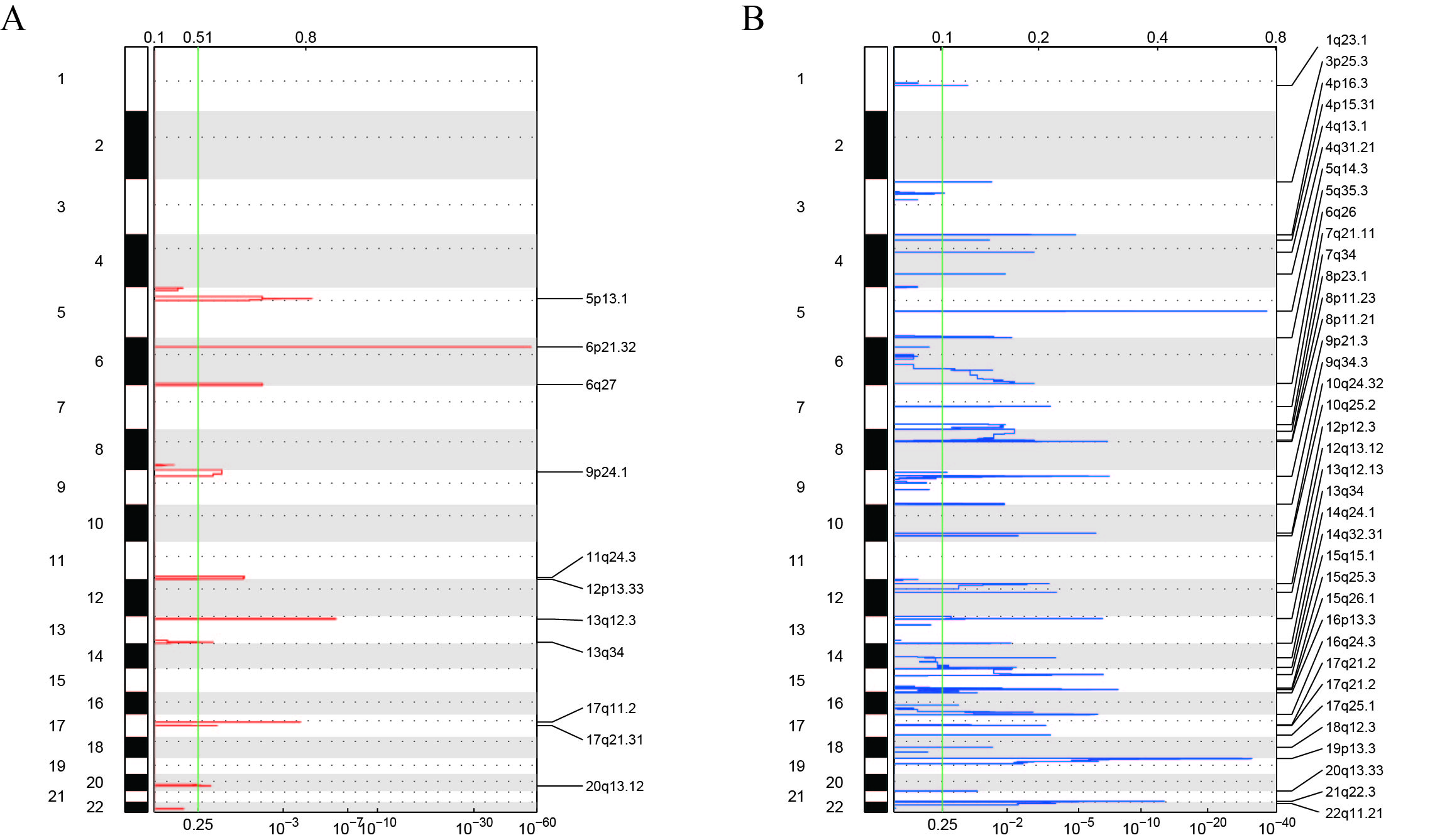

Supplement: Supplementary file 2 — Supplementary Fig. 2: Copy number variations of the top 30 mutated genes. (A) Distribution of the copy number amplification. (B) Distribution of the copy number deletion. [file 13000_2023_1349_MOESM2_ESM.jpg]

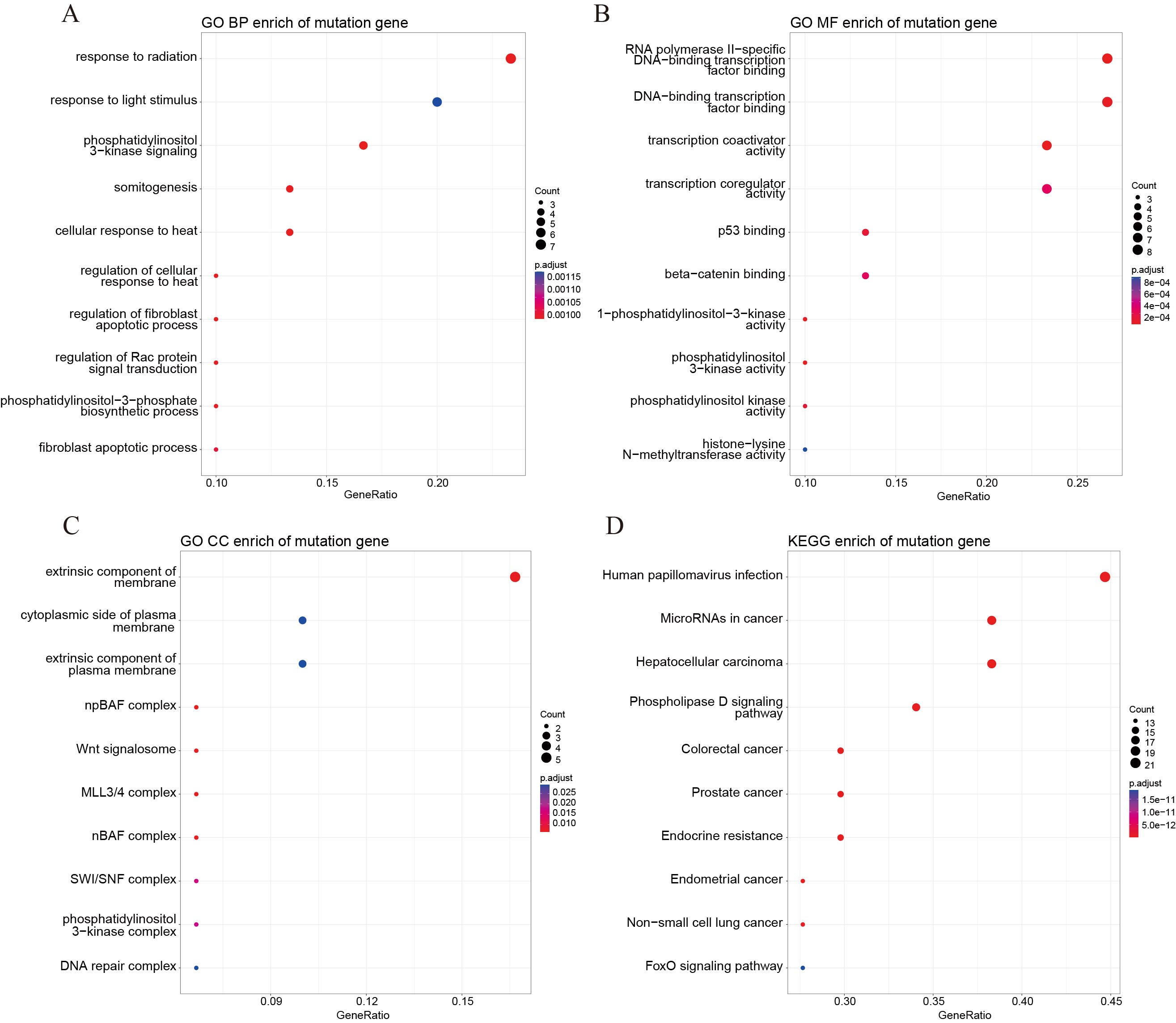

Supplement: Supplementary file 3 — Supplementary Fig. 3: Go and KEGG enrichment analysis of the top 30 mutated genes. (A) Biological process analysis. (B) Molecular function analysis. (C) Cellular component analysis. (D) KEGG enrichment analysis. [file 13000_2023_1349_MOESM3_ESM.jpg]

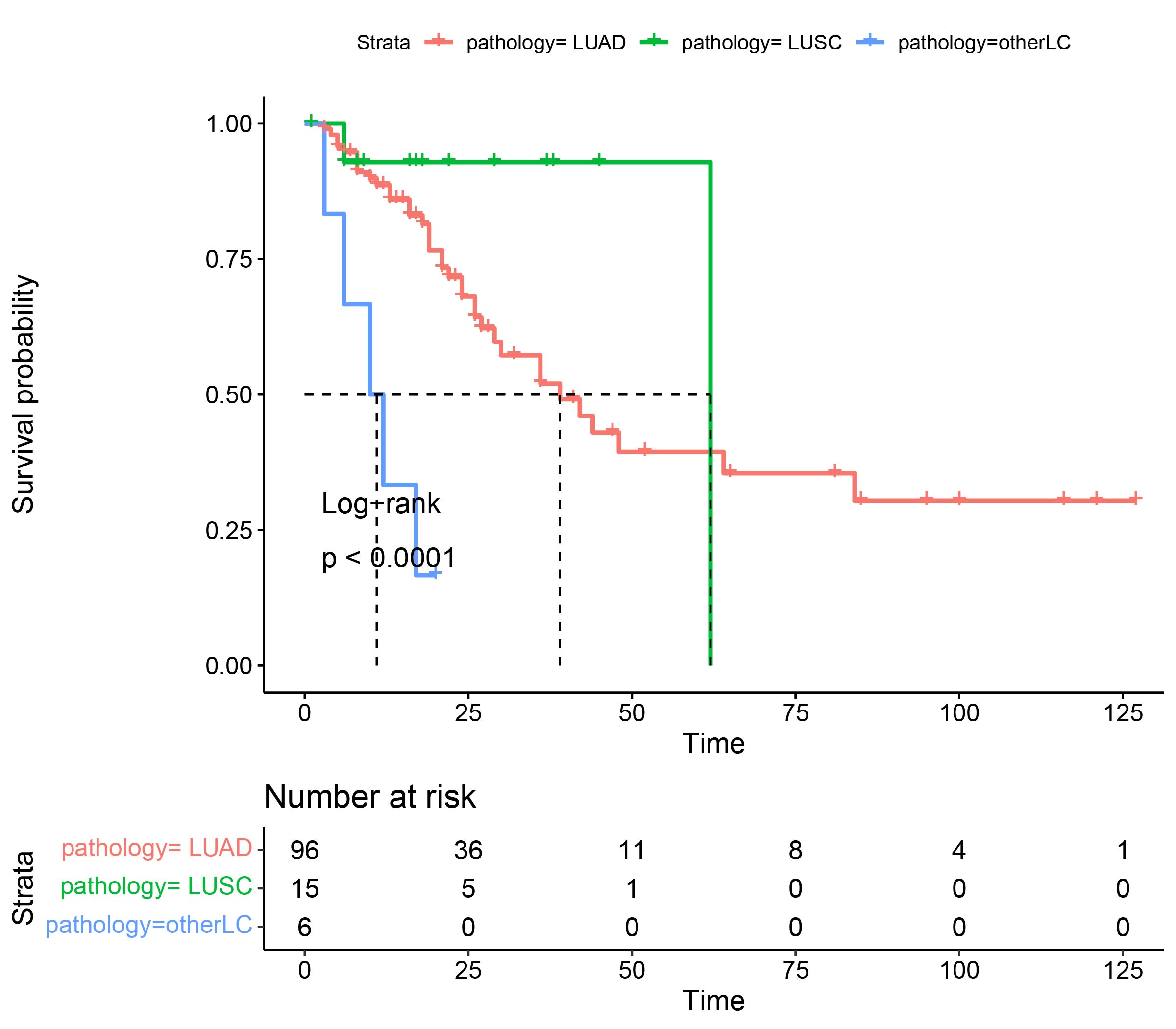

Supplement: Supplementary file 4 — Supplementary Fig. 4: Overall survival analysis of the tumor type. [file 13000_2023_1349_MOESM4_ESM.jpg]
