# Supplementary material for: Molecular profiling and prognostic biomarkers in chinese non-small cell lung cancer cohort
Source: Diagn Pathol. 2023 Jun 10;18:71. doi: 10.1186/s13000-023-01349-1 (PMC10257305; doi:10.1186/s13000-023-01349-1)
Supplement: Supplementary file 5 — Supplementary Table 1: Multivariate Cox proportional hazard analyses of clinicopathological factors for OS in NSCLC cohort. [file 13000_2023_1349_MOESM5_ESM.doc]

**Table S1. Multivariate Cox proportional hazard analyses of clinicopathological factors for OS in NSCLC cohort.**

| **Features** | **Group** | **Univariate** | |  | | **Multivariate** | | |
| --- | --- | --- | --- | --- | --- | --- | --- | --- |
|  |  | HR with 95% CI | *P* Value | |  | | HR with 95% CI | *P* Value |
| Gender | Female | Reference |  |  | |  | |  |
|  | Male | 1.03 (0.55-1.91) | 0.94 |  | |  | |  |
| Smoking | Nonsmoker | Reference |  |  | |  | |  |
|  | Smoker | 1.58 (0.86-2.92) | 0.14 |  | | 6.58 (1.35-32.21) | | 0.02 |
| Pathology | LUAD  LUSC | Reference  0.40 (0.10-0.66) | 0.21 |  | | 0.02 (0-0.23) | | < 0.01 |
| Stage | I-II | Reference |  |  | |  | |  |
|  | III-IV | 1.91 (0.58-0.83) | 0.28 |  | |  | |  |
| TMB | TMB ≤ 3.6  TMB > 3.6 | Reference  1.96 (1.06-3.63) | 0.03 |  | | / | | / |
| *TP53* | Wild | Reference |  |  | |  | |  |
|  | Mutated | 2.34 (1.26-4.35) | 0.007 |  | | / | | / |
| *LRP1B* | Wild | Reference |  |  | |  | |  |
|  | Mutated | 1.53 (0.64-3.64) | 0.34 |  | |  | |  |
| *ZFHX3* | Wild | Reference |  |  | |  | |  |
|  | Mutated | 1.47 (0.45-4.78) | 0.52 |  | |  | |  |
| *PREX2* | Wild | Reference |  |  | |  | |  |
|  | Mutated | 5.23 (1.79-15.29) | 0.003 |  | | 30.5 (5.62-165.4) | | < 0.01 |
| *GRM3* | Wild | Reference |  |  | |  | |  |
|  | Mutated | 1.71 (0.53-5.54) | 0.37 |  | |  | |  |
| *ARID1A* | Wild | Reference |  |  | |  | |  |
|  | Mutated | 8.68 (3.20-23.58) | 0 |  | | 11.02 (2.66-45.64) | | < 0.01 |
| *JAK3* | Wild | Reference |  |  | |  | |  |
|  | Mutated | 1.72 (0.41-7.18) | 0.46 |  | |  | |  |
| *PTPRT* | Wild | Reference |  |  | |  | |  |
|  | Mutated | 6.51 (2.25-18.83) | < 0.01 |  | | / | | / |
| *APC* | Wild | Reference |  |  | |  | |  |
|  | Mutated | 2.34 (0.71-7.63) | 0.16 |  | | 0.14 (0.002-0.8) | | 0.03 |
| *PIK3CG* | Wild | Reference |  |  | |  | |  |
|  | Mutated | 3.4 (1.02-11.26) | 0.046 |  | | 4.28 (1.07-21.7) | | 0.04 |
